# Supplementary material for: DEN-Induced Rat Model Reproduces Key Features of Human Hepatocellular Carcinoma
Source: Cancers (Basel). 2021 Oct 4;13(19):4981. doi: 10.3390/cancers13194981 (PMC8508319; doi:10.3390/cancers13194981)
Supplement: Supplementary file 1 [file cancers-13-04981-s001.zip › cancers-1377242-supplementary.pdf]

Article

DEN-Induced Rat Model Reproduces Key Features of Human Hepatocellular Carcinoma

Keerthi Kurma, Olivier Manches, Florent Chuffart, Nathalie Sturm, Khaldoun Gharzeddine, Jianhui Zhang, Marion Mercey-Ressejac, Sophie Rousseaux, Arnaud Millet, Herve Lerat, Patrice N. Marche, Zuzana Macek Jilkova and Thomas Decaens

Table S1. List of Primers sequences for qPCR analysis.

Gene	Reverse Sequence (5′-3′)	Forward Sequence (5′-3′)
CYCLIN D1	GGCTCCAGAGACAAGAAACG	GCGTACCCTGACACCAATCT
COL1	CTTCTGGGCAGAAAGGACAG	GCCAAGAAGACATCCCTGAA
α-SMA	CATCTCCAGAGTCCAGCACA	ACTGGGACGACATGGAAAAG
TGF-β1	TGGGACTGATCCCATTGATT	ATACGCCTGAGTAGCTGTCT
TIMP1	TGGCTGAACAGGGAAACACT	CAGCAAAAGGCCTTCGTAAA
MMP2	GGGTTTCTTCTGGCTCAGG	TCTGGCTATCCACAAGACTGG
MMP9	GGAAAAGGAAGGAGGGTACG	CCACTCAGGGCCTTCAGAC
CD4	AGAATAGGATGCAGAGCCCC	AAGGCTCCTTCTTCCCAGTC
CD8	CCAATCCCATTCCCTCCACT	TTCTGTCGCTGAACCTGCTA
GAPDH	TTCAGCTCTGGGATGACCTT	CTCATGACCACAGTCCATGC

Table S2. Clinical and biological analysis.

Heading	0 w<br>0 Weeks – Before DEN	8 w<br>8 Weeks of DEN inj	14 w<br>14 weeks of DEN inj	20 w<br>14 Weeks DEN inj + 6 Weeks no DEN
Albumin (g/dL)	3.23 ± 0.04	3.24 ± 0.04	3.16 ± 0.03	3.37 ± 0.03
AST (U/L)	82.2 ± 8.68	69.6 ± 2.38	269 ± 82.5	205.4 ± 59.5
ALT (U/L)	53.2 ± 1.9	53.6 ± 3.1	165.2 ± 44.2$^{*, \#}$	104.4 ± 29.4
GGT (U/L)	1.0 ± 0.0	1.0 ± 0.0	10.5 ± 1.6$^{****, \#\#\#\#}$	3.2 ± 0.7
PT (s)	44.7 ± 14.3	21.5 ± 1.0	24.9 ± 3.4	36.8 ± 16.6
Total Bilirubin (mg/dL)	0.17 ± 0.02	0.23 ± 0.01	0.42 ± 0.02$^{****, \#\#\#\#}$	0.23 ± 0.01
Creatinine (mg/dL)	0.20 ± 0.00	0.30 ± 0.00****	0.37 ± 0.02$^{****, \#\#\#}$	0.39 ± 0.01$^{****, \#\#\#\#}$
Liver fibrosis, METAVIR score; F0/F1/F2/F3/F4 %	F0: 89%<br>F1: 11%	F1: 56%<br>F2: 33%<br>F3: 11%	F2: 44%<br>F3: 22%<br>F4: 33%	F1: 22%<br>F2: 44%<br>F3: 33%

Clinical and biological analysis: AST, aspartate aminotransferase; ALT, alanine aminotransferase; GGT, Gamma-glutamyl transpeptidase; PT, Prothrombin time; Values are means ± SE. Significant difference compared to 0 w; *: $p < 0.05$; **: $p < 0.01$; ***: $p < 0.001$; ****: $p < 0.0001$. Significant difference between 8 w and 14 w; ###: $p < 0.001$; ####: $p < 0.0001$. w = week.

Table S3. Top 25 over-expressed genes in human HCC based using UALCAN cancer database and the fold change observed in DEN-induced tumors of rat model of HCC.

Top 1-25	Gene	Human HCC			DEN-Induced Rat Model of HCC			
		Tumor vs. Normal		Unfavorable Prognosis	14w Tumor vs. 14w No DEN		20 w Tumor vs. 20 w NoDEN	
		Fold Change	*p*-value	*p*-value	Fold Change	*p*-Value	Fold Change	*p*-value
1	GPC3	94.5	<0.0001	*ns*	14.1	<0.0001	13.5	<0.0001
2	LCN2	36.0	<0.0001	*ns*	4.1	0.0180	6.5	0.0347
3	SPP1	34.3	<0.0001	*<0.0001*	6.6	<0.0001	6.5	<0.0001
4	UBE2C	33.1	<0.0001	*<0.0001*	8.2	<0.0001	4.4	<0.0001
5	PTTG1	26.2	<0.0001	*<0.0001*	7.2	<0.0001	4.9	<0.0001
6	SFN	24.2	<0.0001	*<0.0001*	NA		NA	
7	MDK	20.7	<0.0001	*0.0057*	2.0	0.0311	0.9	ns
8	UBE2T	18.6	<0.0001	*<0.0001*	9.2	<0.0001	5.2	<0.0001
9	CCNB1	17.8	<0.0001	*<0.0001*	10.0	<0.0001	6.1	<0.0001
10	AKR1B10	16.1	<0.0001	*0.0022*	229.8	<0.0001	70.4	<0.0001
11	NDUFA4L2	13.8	<0.0001	*0.0230*	2.4	ns	1.6	ns
12	NT5DC2	11.7	<0.0001	*<0.0001*	4.3	<0.0001	4.1	<0.0001
13	PLVAP	11.6	<0.0001	*0.0042*	2.3	<0.0001	2.0	<0.0001
14	G6PD	11.1	<0.0001	*<0.0001*	3.6	<0.0001	3.2	0.0002
15	PDZK1IP1	11.1	<0.0001	*0.2300*	3.9	0.0017	4.2	0.0015
16	CENPW	10.6	<0.0001	*<0.0001*	10.2	<0.0001	3.8	<0.0001
17	SPARCL1	10.6	<0.0001	*0.0016*	1.4	ns	1.4	ns
18	SPINK1	10.1	<0.0001	*0.0083*	0.5	ns	0.5	ns
19	UBD	9.2	<0.0001	*0.1600*	3.1	<0.0001	2.9	<0.0001
20	THY1	9.1	<0.0001	*0.0014*	22.6	<0.0001	30.8	<0.0001
21	PTP4A3	8.9	<0.0001	*0.0360*	1.8	0.0004	2.0	0.0002
22	TK1	8.7	<0.0001	*<0.0001*	3.1	<0.0001	1.8	0.0001
23	TACC3	8.0	<0.0001	*<0.0001*	4.2	<0.0001	3.3	<0.0001
24	GMNN	7.4	<0.0001	*0.0029*	3.1	<0.0001	1.6	0.0057
25	STMN1	7.4	<0.0001	*<0.0001*	5.1	<0.0001	3.8	<0.0001

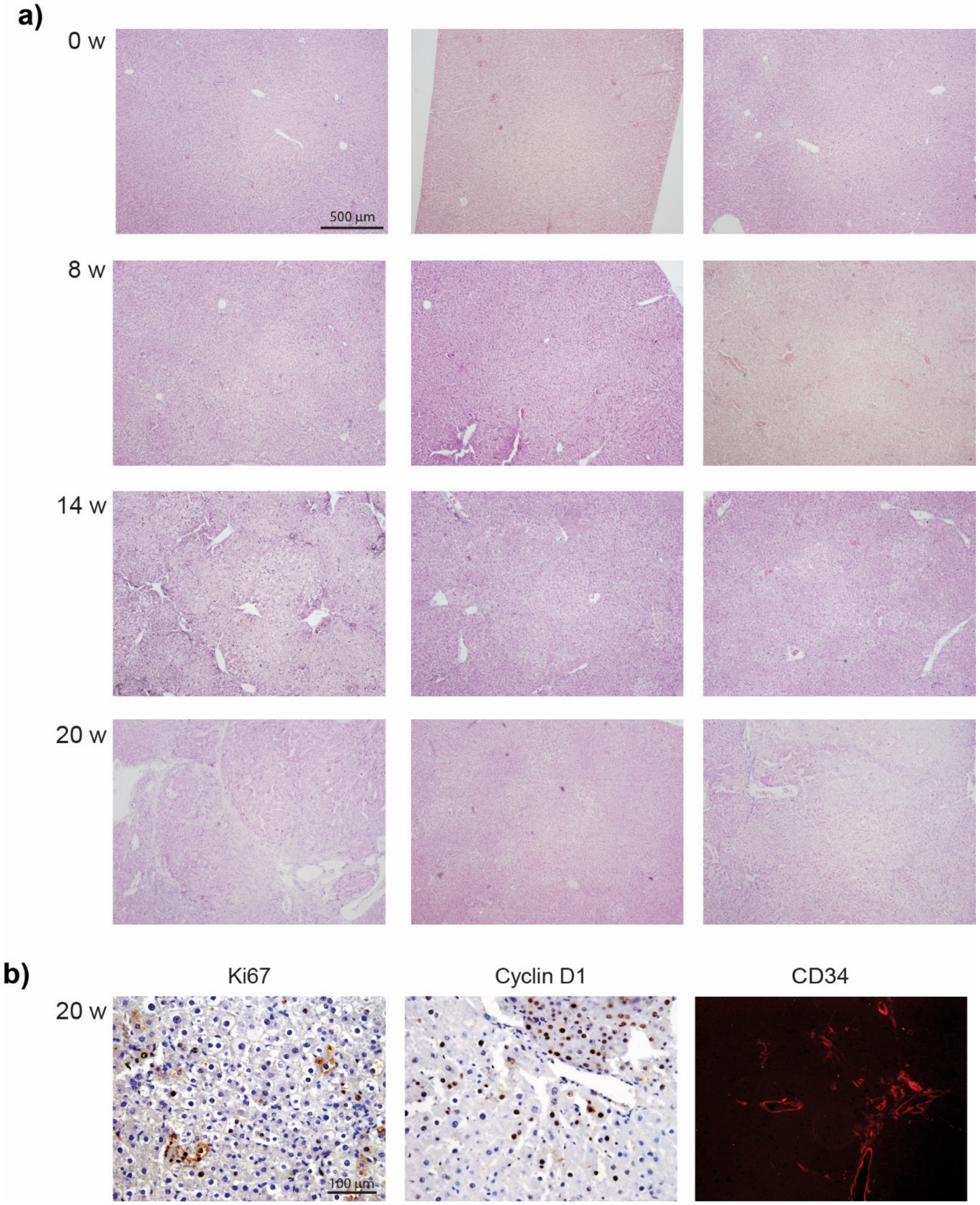

Figure S1. (**a**) Representative images of hematoxylin and eosin (H&E)-stained liver tissue sections (4× magnification). (**b**) Representative images of nuclear Ki67, CyclinD1 (20× magnification) and CD34 staining in 20 weeks group (4× magnification), w = weeks.

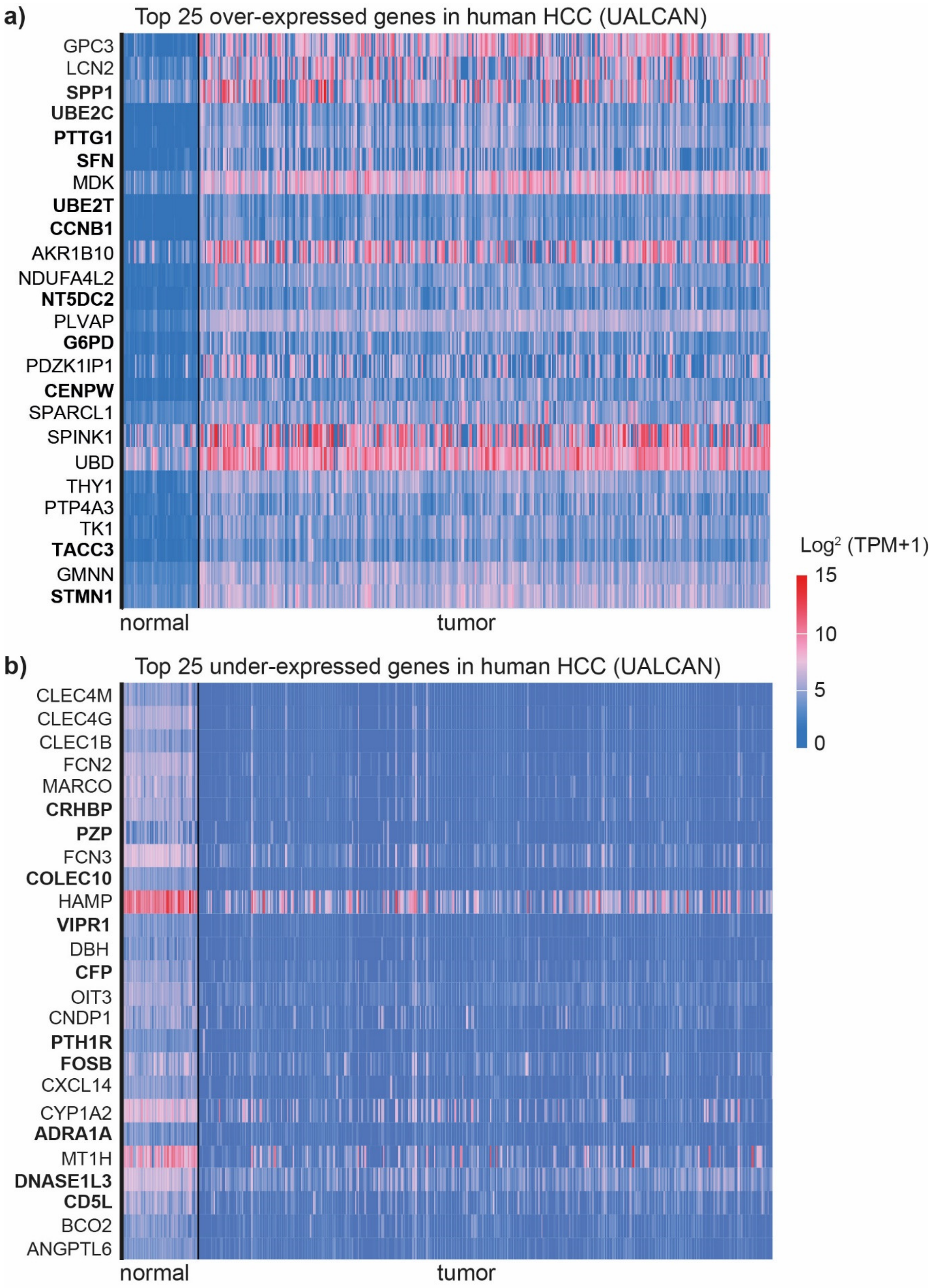

Figure S2. Heatmaps showing the relative expression of top 25 over-expressed (**a**) and top 25 under-expressed genes (**b**) in human HCC based using UALCAN cancer database.

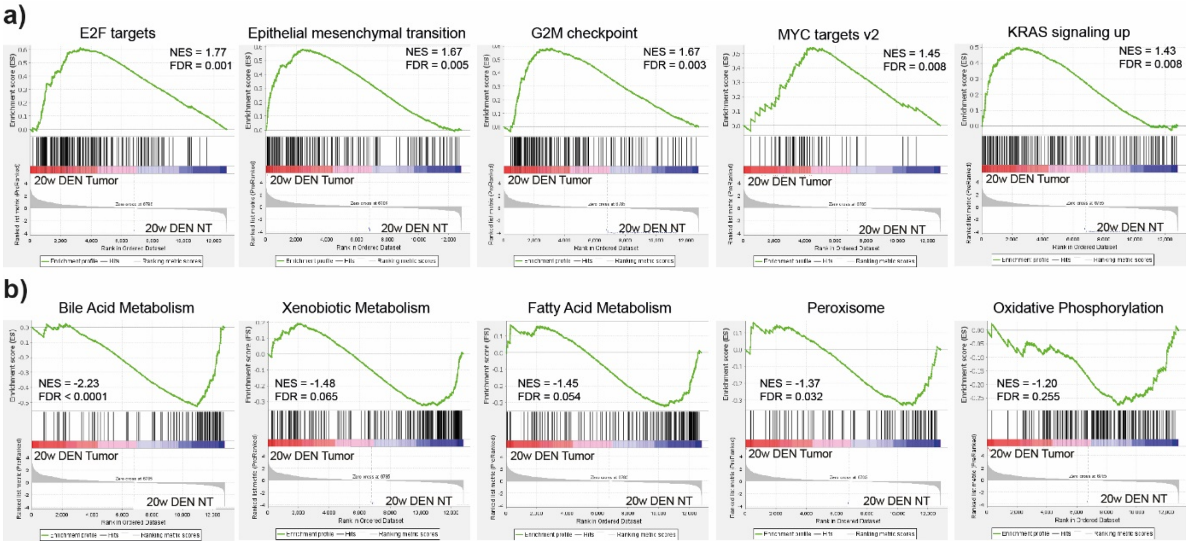

Figure S3. Gene set enrichment analysis of tumor tissue versus non-tumoral tissue in DEN treated rat 20 weeks group (14 weeks of DEN injections + 6 weeks no DEN). (**a**) Top 5 positively enriched gene sets, (**b**) Top 5 depleted gene sets. NES, normalized enrichment score; FDR, false discovery rate.

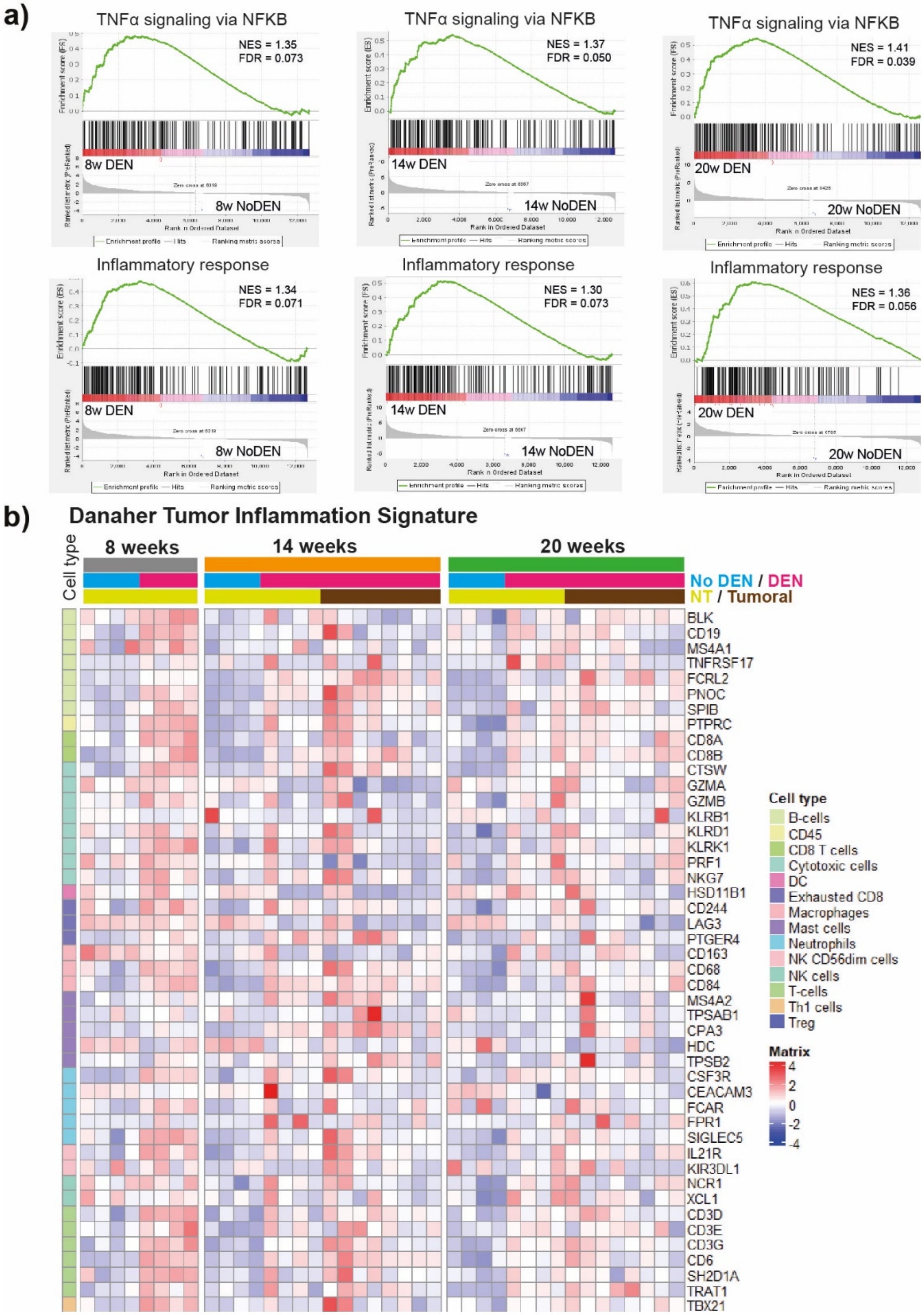

Figure S4. (**a**) Gene set enrichment analysis of DEN treated rat versus no DEN treated rats of the same age. NES, normalized enrichment score; FDR, false discovery rate. (**b**) Heatmap showing the relative expression of genes from tumor inflammatory signature [1]. Subclasses are stratified based on lymphocyte infiltration and on the activation of either immunosuppressive or pro-inflammatory signals.

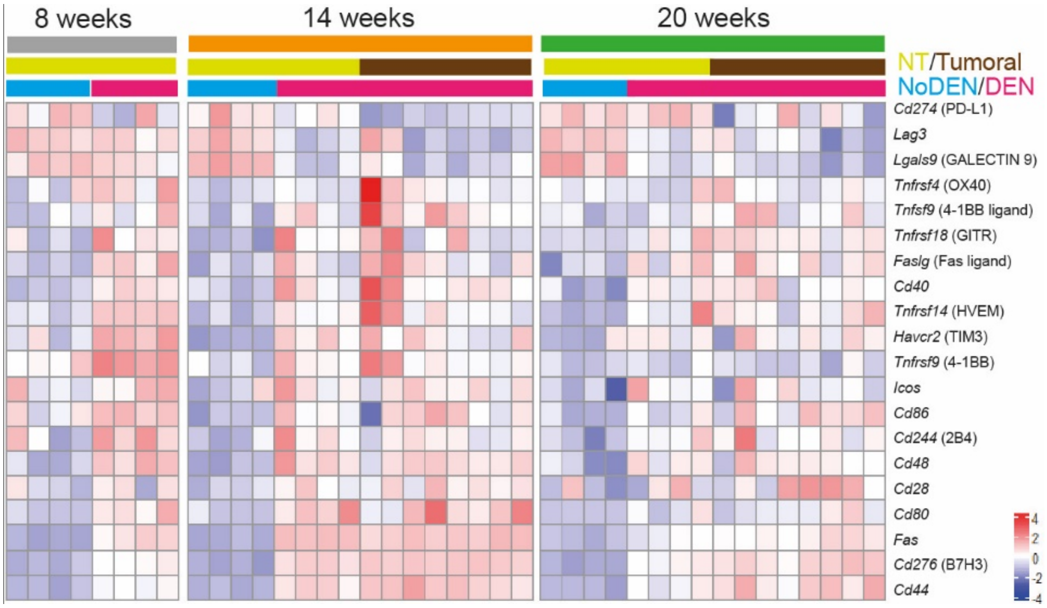

Figure S5. Heatmap showing the relative expression of genes for main immune checkpoint molecules.

References:

1. Moeini, A.; Torrecilla, S.; Tovar, V.; Montironi, C.; Andreu-Oller, C.; Peix, J.; Higuera, M.; Pfister, D.; Ramadori, P.; Pinyol, R., et al. An Immune Gene Expression Signature Associated With Development of Human Hepatocellular Carcinoma Identifies Mice That Respond to Chemopreventive Agents. *Gastroenterology* **2019**, *157*, 1383-1397, doi:10.1053/j.gastro.2019.07.028.
